# The association between financial toxicity and mortality in hematologic malignancies: a systematic review and meta-analysis

**DOI:** 10.1093/oncolo/oyag178

**Published:** 2026-05-07

**Authors:** Fang Liao, Qiu-xia Xu, Ping Lin, Jian-qiong Feng, Dan Chen

**Affiliations:** Department of Hematology, Chinese People's Liberation Army, The General Hospital of Western Theater Command, Chengdu, Sichuan 610083, China; Department of Hematology, Chinese People's Liberation Army, The General Hospital of Western Theater Command, Chengdu, Sichuan 610083, China; Department of Hematology, Chinese People's Liberation Army, The General Hospital of Western Theater Command, Chengdu, Sichuan 610083, China; Department of Otorhinolaryngology-Head and Neck Surgery, Chinese People's Liberation Army, The General Hospital of Western Theater Command, Chengdu, Sichuan 610083, China; Department of Hematology, Chinese People's Liberation Army, The General Hospital of Western Theater Command, Chengdu, Sichuan 610083, China

**Keywords:** financial toxicity, hematologic malignancies, mortality, socioeconomic factors, systematic review, meta-analysis

## Abstract

**Background:**

Hematologic malignancies impose substantial economic burdens, but the quantitative association between financial toxicity and overall survival remains poorly defined across diverse clinical settings and disease lineages.

**Methods:**

Adhering to PRISMA and MOOSE guidelines, we searched electronic databases through January 2026 for studies reporting multivariable-adjusted hazard ratios (aHRs) for the association between financial toxicity and mortality in adults diagnosed with hematologic malignancies. A random-effects model with Hartung–Knapp adjustment was used for data synthesis. Small-study effects were addressed via the Duval and Tweedie trim-and-fill method.

**Results:**

Eleven cohorts involving 280 826 individuals were included. Meta-analysis identified a significant association between financial toxicity and inferior survival (pooled aHR: 1.57; 95% CI, 1.29-1.91; *P *< .001). High heterogeneity (*I*^2^ = 85.1%) and a wide 95% prediction interval (0.89-2.77) indicated substantial variability across settings. Subgroup analysis revealed that mortality risk was primarily linked to structural barriers, specifically lack of insurance (aHR: 1.66; 95% CI, 1.39-1.99), whereas this association was weaker in specialized transplant settings with integrated psychosocial support. Findings remained statistically significant after trim-and-fill adjustment for potential publication bias (adjusted aHR: 1.33; 95% CI, 1.05-1.69; *P *= .023).

**Conclusions:**

Financial toxicity is a significant factor independently associated with mortality in hematologic malignancies. The strength of this association varies depending upon the care environment, with structural access barriers representing the highest risk. Integrating proactive financial navigation into standard clinical pathways is essential to alleviate socioeconomic disparities and may help improve survival outcomes.

Implications for Practice

- Clinical Integration: Financial toxicity is a critical factor associated with a substantially increased mortality risk across hematologic malignancies. It should be integrated into the prognostic framework as a standard component of clinical assessment, rather than viewed solely as a quality-of-life concern.
- Prioritizing Structural Barriers: Objective indicators of financial strain, specifically insurance status, which demonstrate a stronger and more consistent correlation with mortality than subjective measures of perceived distress. Clinical interventions should prioritize addressing insurance gaps and resource navigation to ensure uninterrupted access to high-cost therapies.
- Scalable Support Models: The survival parity observed in hematopoietic cell transplantation programs suggests that the mortality risk associated with financial toxicity is modifiable. Expanding the proactive financial navigation and multidisciplinary social support models inherent to hematopoietic cell transplantation programs into general hematology settings may help mitigate socioeconomic disparities in survival.
- Global Generalizability: While the association between financial toxicity and mortality is robust in US-based cohorts, the impact in universal healthcare systems and low-resource settings remains less defined. Prospective validation in diverse global healthcare systems is an urgent priority to confirm the generalizability of these conclusions and inform targeted policy interventions.

## Introduction

Hematologic malignancies, encompassing leukemia, lymphoma, and multiple myeloma, represent a significant and growing global health burden.^1^ Over the past two decades, the therapeutic landscape for these diseases has undergone a major paradigm shift. The advent of targeted therapies, immunotherapies, and next-generation chemotherapies has dramatically improved patient prognosis and prolonged overall survival.^2^ However, these remarkable survival benefits are linked to a substantial economic cost. Characterized by prolonged treatment courses, complex regimens, and high drug prices, hematologic malignancies consistently rank among the most expensive cancers to treat, imposing an unprecedented financial burden on patients, their families, and the broader healthcare system.^3^

This economic hardship associated with diagnosis and treatment is increasingly recognized as “financial toxicity.”^4^ Analogous to physical toxicities, financial toxicity encompasses direct out-of-pocket expenses, indirect costs, such as lost income and employment disruption and associated psychological distress.^5^ The threat of financial toxicity is particularly pronounced in hematologic oncology due to the chronicity and intensity of care. For instance, patients with chronic myeloid leukemia often require lifelong tyrosine kinase inhibitor therapy,^6^ while those with multiple myeloma undergo years of multiline treatments and stem cell transplantation. Financial strain is closely associated with poor treatment adherence, including cost-related delays, skipped treatments, and discontinuation of essential therapies.^7^ Furthermore, this financial barrier limits access to advanced therapeutics, such as CAR-T cell therapies,^8^ potentially contributing to disparities in survival outcomes.

While the impact of financial toxicity on patient-reported outcomes and adherence is established, its association with overall survival remains fragmented. Existing evidence is largely confined to single disease entities, limiting cross-lineage comparisons across hematologic malignancies. In addition, the measurement of financial toxicity is highly variable,^9^ with studies utilizing differing objective socioeconomic proxies or subjective patient-reported measures, complicating data synthesis. This literature is also geographically skewed toward high-income countries, leaving a data void for low- and middle-income countries where catastrophic health expenditures are prevalent and associated with poorer outcomes.^10^

To bridge this critical knowledge gap, we conducted a systematic review and meta-analysis to evaluate the association between financial toxicity and overall survival in individuals with hematologic malignancies. Our primary objective was to quantify the pooled mortality risk associated with severe financial burden. Through subgroup analyses, we explored sources of heterogeneity, specifically examining whether this association varies by malignancy subtype or measurement approach (objective vs subjective). We hypothesized that financial toxicity is significantly associated with inferior survival outcomes, suggesting that structured financial navigation may be relevant as a component of standard clinical management.

## Methods

### Study design and protocol registration

This systematic review and meta-analysis was conducted in accordance with the PRISMA 2020 and MOOSE guidelines.^11^^,^^12^ The protocol was registered with PROSPERO (CRD420261288560) on January 19, 2026. To ensure full transparency, we note that while the initial literature search was completed in early January 2026, protocol registration was finalized strictly prior to data extraction and statistical synthesis.

### Search strategy

A comprehensive and systematic literature search was executed across multiple electronic databases, including PubMed, Embase, Web of Science Core Collection, the Cochrane Library, and CINAHL from database inception to January 2026. The search strategy combined controlled vocabulary and free-text keywords across three core concepts: (1) hematologic malignancies (eg, “Leukemia,” “Lymphoma,” “Multiple Myeloma,” and “Hematologic Neoplasms”); (2) financial toxicity such as “Financial Distress,” “Economic Burden,” “Cost of Illness,” “Cost-related Nonadherence,” “Catastrophic Health Expenditure,” and “Bankruptcy”; and (3) survival outcomes (eg, “Survival Rate,” “Mortality,” “Prognosis,” and “Hazard Ratio”). Additionally, we manually screened the reference lists of included studies and relevant systematic reviews to identify potential eligible articles missed by the primary search.

### Eligibility criteria

Study eligibility was defined using the PICOS framework. Inclusion criteria were: (1) Population: Adults (≥18 years) with hematologic malignancy (including leukemia, lymphoma, or multiple myeloma). (2) Exposure to financial toxicity or its objective proxies, such as socioeconomic status, income level, insurance coverage, or bankruptcy, and compared to groups with lower financial burden. (3) Outcome: Report overall survival or all-cause mortality with hazard ratios and 95% confidence intervals, or sufficient data for their estimation. (4) Study Design: original observational study designs. We excluded nonoriginal research, conference abstracts lacking sufficient data, pediatric studies, and studies without mortality endpoints.

### Study selection and data extraction

Two investigators independently screened titles, abstracts, and full texts. Discrepancies were resolved by consensus or a third senior investigator. Data were independently extracted using a standardized form, capturing study characteristics (author, year, country, design, and data source), patient demographics, malignancy subtypes, and financial toxicity definitions. For survival outcomes, we extracted the most comprehensively adjusted multivariable hazard ratios and their 95% confidence intervals to minimize the impact of clinical confounders. Specifically, from the study by Fiala et al.,^13^ only the multicenter institutional cohort was included, while the SEER-based cohort was excluded to prevent patient population overlap with other SEER-based studies (eg, Huang et al.^14^) included in this meta-analysis.

### Quality assessment

Two investigators independently assessed methodological quality using the Newcastle-Ottawa Scale for cohort studies.^15^ Studies were scored ranging from 0 to 9 stars as high quality (7-9 stars), moderate quality (4-6 stars), or low quality (0-3 stars). Scoring discrepancies were resolved through consensus or via consultation with a senior author.

### Statistical analysis

Hazard ratios and 95% CIs for all-cause mortality were log-transformed to stabilize variance, and then pooled using a random-effects model with the restricted maximum-likelihood estimator.

Statistical heterogeneity was assessed using Cochran’s *Q*-test and the *I*^2^ statistic, with *I*^2^ > 50% or a *P *< .10 for the *Q*-test indicating of substantial heterogeneity. We performed pre-specified subgroup analyses stratified by disease lineage and measurement dimension.

The robustness was evaluated through leave-one-out sensitivity analyses. Publication bias was assessed by visual inspection of funnel plots and formally quantified using Egger’s linear regression test. All analyses were performed using *R version 4.5.2*, with the '*meta*' and '*metafor'* packages. A two-sided *P *< .05 was defined as the threshold for statistical significance, with the exception of the *Q*-test for heterogeneity (*P *< .10).

## Results

### Study selection and characteristics

**Figure 1.**
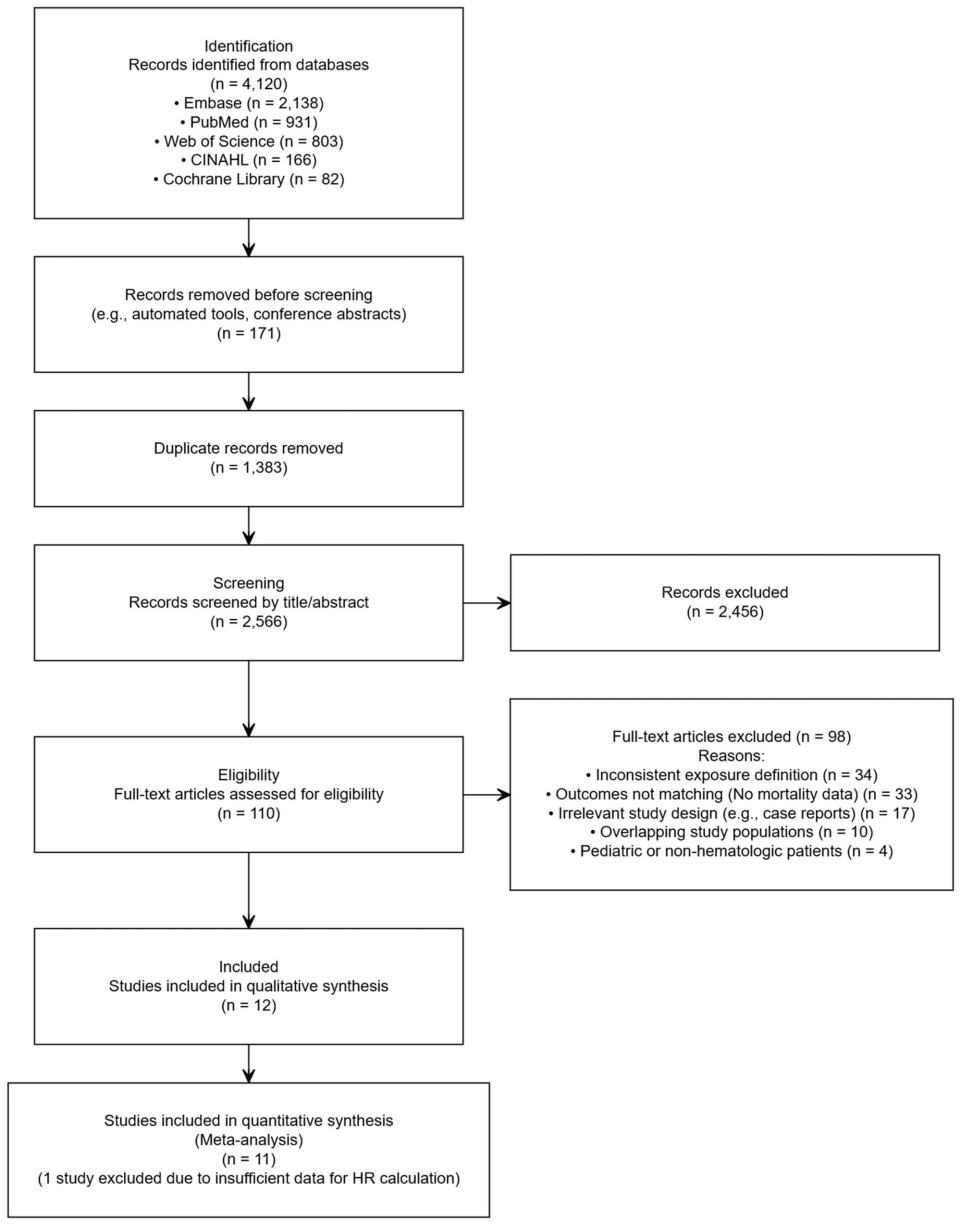
PRISMA 2020 flow diagram of the study selection process.

Our systematic search identified 4120 records. After the removal of 171 conference abstracts and 1383 duplicate citations, 2566 unique records were screened by title and abstract, excluding 2456 irrelevant studies. Of 110 articles undergoing full-text evaluation, 98 articles were excluded, primarily due to incorrect exposure or outcome definitions (detailed in Figure 1).

Ultimately, 12 studies met eligibility criteria for qualitative systematic review. For the quantitative synthesis, 11 studies provided extractable hazard ratios and 95% confidence intervals required for pooling.^13^^,^^14^^,^^16–24^ One study^25^ was excluded from the primary meta-analysis due to its high risk of bias and extreme estimates, as further validated in our sensitivity analysis. The comprehensive clinical and demographic characteristics are detailed in Table 1.

**Table 1.** Clinical and methodological characteristics of included studies (*N *= 12).

Study (year)	Country/region (source)	Design	Malignancy type	Sample size (*N*)	FT measure (Exposure vs Ref)	Follow-up (Mos)	Outcome	Key adjusted factors
**Hussaini et al. (2025)**	USA (Alabama)	Prospective	BMT (Heme)	214	COST score	12	OS	Age, income, education
**Khera et al. (2018)**	USA (Multicenter)	Prospective	HCT (Heme)	325	Financial Hardship	24	OS	Age, HCT type, income, education
**Huang et al. (2021)**	USA (SEER)	Retrospective	MM	17 981	Insurance	120	OS, CSS	Age, sex, race, SES
**Tao et al. (2014)**	USA (California)	Retrospective	DLBCL	33 032	nSES	54 (med)	OS, CSS	Treatment, stage, age, B-symptoms
**Goldstein et al. (2018)**	USA (NCDB)	Retrospective	FL	43 648	Insurance	58 (med)	OS	Age, sex, race, stage, comorbidity
**Perry et al. (2017)**	USA (SEER)	Retrospective	CML	3626	Insurance	32 (med)	OS	Age, race, sex, county SES, marital
**Chohan et al. (2022)**	USA (NCDB)	Retrospective	WM	1249	Insurance	56 (med)	OS	Age, sex, race, comorbidity, SES
**Chamoun et al. (2021)**	USA (NCDB)	Retrospective	MM	117 926	Insurance & Income	120	OS	Age, income, comorbidity, facility
**Fiala et al. (2015)**	USA (WUSM/SEER)	Retrospective	MM	46 067	SES & Insurance	24-49	OS	Age, race, comorbidity, SCT use
**Wang et al. (2017)**	USA (SEER)	Retrospective	HL	14 286	Insurance	96 (max)	OS	Race, sex, histology, stage, SES
**Nnawuba et al. (2023)**	USA (Arkansas)	Retrospective	Mixed Heme	2472	Insurance	NR	OS	Age, sex, race
**Nethala et al. (2025)**	India (Single)	Retrospective	AML	29^a^	SES (Kuppuswamy)	8 (med)	OS	Age, education, income, support

Exposure groups represent the cohort with higher financial toxicity or economic disadvantage (eg, uninsured, Medicaid-insured, or lowest income quintile/SES), while reference groups represent those with higher economic advantage (eg, privately insured or highest income quintile/SES).

a The sample size (*n* = 29) refers to the final analyzed cohort of patients who received definitive treatment and had complete follow-up data at the study center, whereas the total initial enrollment was 41.

Abbreviations: aHR, adjusted hazard ratio; AML, acute myeloid leukemia; BMT, bone marrow transplant; CI, confidence interval; CML, chronic myeloid leukemia; COST, Comprehensive Score for Financial Toxicity; CSS, cancer-specific survival; DLBCL, diffuse large B-cell lymphoma; FL, follicular lymphoma; FT, financial toxicity; HCT, hematopoietic cell transplantation; HL, Hodgkin lymphoma; HR, hazard ratio; med, median; MHI, median household income; mPS, multiple propensity score; NCDB, National Cancer Database; NR, not reported; nSES, neighborhood socioeconomic status; OS, overall survival; SCT, stem cell transplant; SEER, Surveillance, Epidemiology, and End Results; SES, socioeconomic status; WM, Waldenström macroglobulinemia; WUSM, Washington University School of Medicine.

The included studies encompassed a broad clinical spectrum: multiple myeloma (n = 3), various lymphoma subtypes (DLBCL, FL, and HL; *n* = 3), myeloid leukemias (*n* = 3), and mixed hematologic or transplant cohorts. Geographically, 11 investigations were conducted in the United States, predominantly utilizing national registries such as the Surveillance, Epidemiology, and End Results program and the National Cancer Database, while one was in a developing economy (India). Methodologically, 10 were retrospective cohorts, while two were prospective longitudinal studies focusing on transplant settings.

### Assessment of financial toxicity and mortality

Financial toxicity definitions were heterogeneous. As summarized in Table 1, eight studies utilized objective proxies, primarily health insurance status (eg, uninsured or Medicaid-insured vs privately insured) or neighborhood-level socioeconomic status quintiles. Conversely, four studies employed validated patient-reported outcome measures or structured surveys, such as the Comprehensive Score for Financial Toxicity or dedicated financial hardship questionnaires, to capture the subjective experience of economic strain.

Overall survival was the primary endpoint, with two studies additionally reporting cancer-specific survival. Median follow-up durations were generally robust, typically exceeding 32 months in registry-based cohorts; however, substantially shorter follow-up (median 8 days) was observed in small-scale clinical studies. All but conference abstracts provided hazard ratios adjusted for critical confounders, including age, disease stage, and comorbidities.

### Quality assessment and risk of bias

The individual scores and quality gradings are presented in Table 2. The evidence base was generally of high-to-moderate quality, with the Newcastle-Ottawa Scale scores ranging from 4 to 9 stars. Seven studies were categorized as high quality (score ≥ 8); four studies, published as conference abstracts, were rated as moderate quality (score 6-7) due to constrained reporting on methodology and follow-up completeness. One single-center study was classified as low quality (score 4) due to its limited analytical sample (*N *= 29) and an elevated risk of attrition bias (29.3% loss-to-follow-up). These assessments informed subsequent sensitivity analyses regarding study quality.

**Table 2.** Quality assessment using the Newcastle-Ottawa Scale (NOS).

Study (year)	Study design	Selection (Max 4★)	Comparability (Max 2★)	Outcome (Max 3★)	Total score	Quality level
**Hussaini et al. (2025)**	Prospective Cohort^a^	★★★	★	★★	6	Moderate
**Khera et al. (2018)**	Prospective Cohort	★★★★	★★	★★★	9	High
**Huang et al. (2021)**	Retrospective (SEER)	★★★★	★★	★★★	9	High
**Goldstein et al. (2018)**	Retrospective (NCDB)	★★★★	★★	★★★	9	High
**Perry et al. (2017)**	Retrospective (SEER)	★★★★	★★	★★★	9	High
**Tao et al. (2014)**	Retrospective (Registry)	★★★★	★★	★★★	9	High
**Fiala et al. (2015)**	Retrospective (Single+SEER)	★★★	★★	★★★	8	High
**Chohan et al. (2022)**	Retrospective (NCDB)	★★★★	★★	★★★	9	High
**Wang et al. (2017)**	Retrospective (SEER)^a^	★★★	★	★★★	7	Moderate
**Nnawuba et al. (2023)**	Retrospective (Registry)^a^	★★★	★	★★	6	Moderate
**Nethala et al. (2025)**	Retrospective (Single-center)	★★	★	★	4	Low
**Chamoun et al. (2021)**	Retrospective (NCDB)	★★★★	★	★★★	8	High

a Indicates studies published as conference abstracts, which were rated lower due to limited details on methodology and participant follow-up.

### Meta-analysis results

The primary quantitative synthesis examined the association between financial toxicity and overall survival. To ensure consistency across heterogeneous exposure definitions, all effect estimates were standardized to represent the mortality risk of the high financial toxicity group (eg, uninsured, Medicaid-insured, or lowest income quartile) relative to the low financial toxicity reference group (eg, privately insured or highest income quartile). Study-level data, including specific comparisons and subgroup classifications, are summarized in Table 3.

**Table 3.** Summary of adjusted hazard ratios for mortality associated with financial toxicity.

Malignancy subgroup	Study (year)	Exposure category	Comparison (high FT vs Ref)	aHR (95% CI)	Analysis use
**Plasma cell & WM**	Chamoun (2021)	Insurance Status	Uninsured vs Private	1.62 (1.32-1.99)	Primary
	Chamoun (2021)	Insurance Status	Medicaid vs Private	1.59 (1.36-1.87)	Subgroup
	Huang (2021)	Insurance Status	Uninsured vs Private	1.33 (1.20-1.48)	Primary
	Huang (2021)	Insurance Status	Medicaid vs Private	1.67 (1.56-1.78)	Subgroup
	Fiala (2015)	SES	Low vs High SES	1.54 (1.13-2.09)	Primary
	Chohan (2022)	Insurance Status	Uninsured vs Private	3.11 (1.77-5.46)	Primary
	Chohan (2022)	Insurance Status	Medicaid vs Private	1.88 (1.01-3.48)	Subgroup
**Lymphoma**	Goldstein (2018)	Insurance Status	Uninsured vs Private	1.96 (1.69-2.28)	Primary
	Goldstein (2018)	Insurance Status	Medicaid vs Private	1.83 (1.57-2.12)	Subgroup
	Wang (2017)	Insurance Status	Medicaid vs Private	2.37 (1.56-3.60)	Primary
	Wang (2017)	Insurance Status	Uninsured vs Private	2.26 (1.35-3.80)	Subgroup
	Tao (2014)	nSES	Lowest vs Highest Quintile	1.37 (1.26-1.48)	Primary
**Leukemia**	Perry (2017)	Insurance Status	Uninsured vs Private	1.93 (1.40-2.66)	Primary
	Perry (2017)	Insurance Status	Medicaid vs Private	1.83 (1.39-2.40)	Subgroup
	Nethala (2025)^a^	SES	Lower-mid vs Upper SES	19.90 (1.39-286.0)	Sensitivity^a^
**Transplant/mixed**	Hussaini (2025)	Perceived FT	High vs Low FT (COST)	1.14 (0.63-2.07)	Primary
	Khera (2018)	Perceived FT	Financial Hardship: Yes vs No	1.00 (0.88-1.14)^b^	Primary
	Nnawuba (2023)	Insurance Status	Medicaid vs Private	1.48 (1.19-1.84)^c^	Primary

Exposure groups represent the cohort with higher financial toxicity or economic disadvantage (eg, uninsured, Medicaid-insured, or lowest income quintile), while reference groups represent those with higher economic advantage (eg, privately insured or highest income quintile).

Definitions: Primary: Estimate included in the main meta-analysis. Subgroup: Estimate utilized specifically for insurance-type or malignancy-specific subgroup analyses. Sensitivity: Estimate excluded from primary pooling due to extreme variance or high bias risk but included in robustness checks.

Study-specific clarifications:

a Nethala et al. (2025): Due to extreme variance (95% CI, 1.39-286.0) and a high risk of small-study bias, this study was included in the qualitative synthesis but excluded from the primary pooled analysis to preserve statistical stability and prevent the artificial inflation of heterogeneity (*I*^2^). Its impact was further evaluated in sensitivity analyses.

b Khera et al. (2018): A conservative hazard ratio (HR) of 1.00 was imputed for this study, aligning with the primary authors reported “lack of impact on survival” and consistent nonsignificant findings (*P *> .05) across all financial hardship domains. This approach was adopted to minimize potential overestimation bias in the overall pooled effect.

c Nnawuba et al. (2023): As adjusted hazard ratios were not explicitly reported, the effect size was derived from published survival data according to the comprehensively updated guidance and practical methods described by Tierney et al.^26^ to ensure comparability with the other registry-based cohorts.

Abbreviations: aHR, adjusted hazard ratio; AML, acute myeloid leukemia; BMT/HCT, bone marrow/hematopoietic cell transplant; CI, confidence interval; CML, chronic myeloid leukemia; DLBCL, diffuse large B-cell lymphoma; FL, follicular lymphoma; HL, Hodgkin lymphoma; MM, multiple myeloma; nSES, neighborhood socioeconomic status; SES, socioeconomic status; WM, Waldenström macroglobulinemia.

### Meta-analysis of financial toxicity and mortality

**Figure 2.**
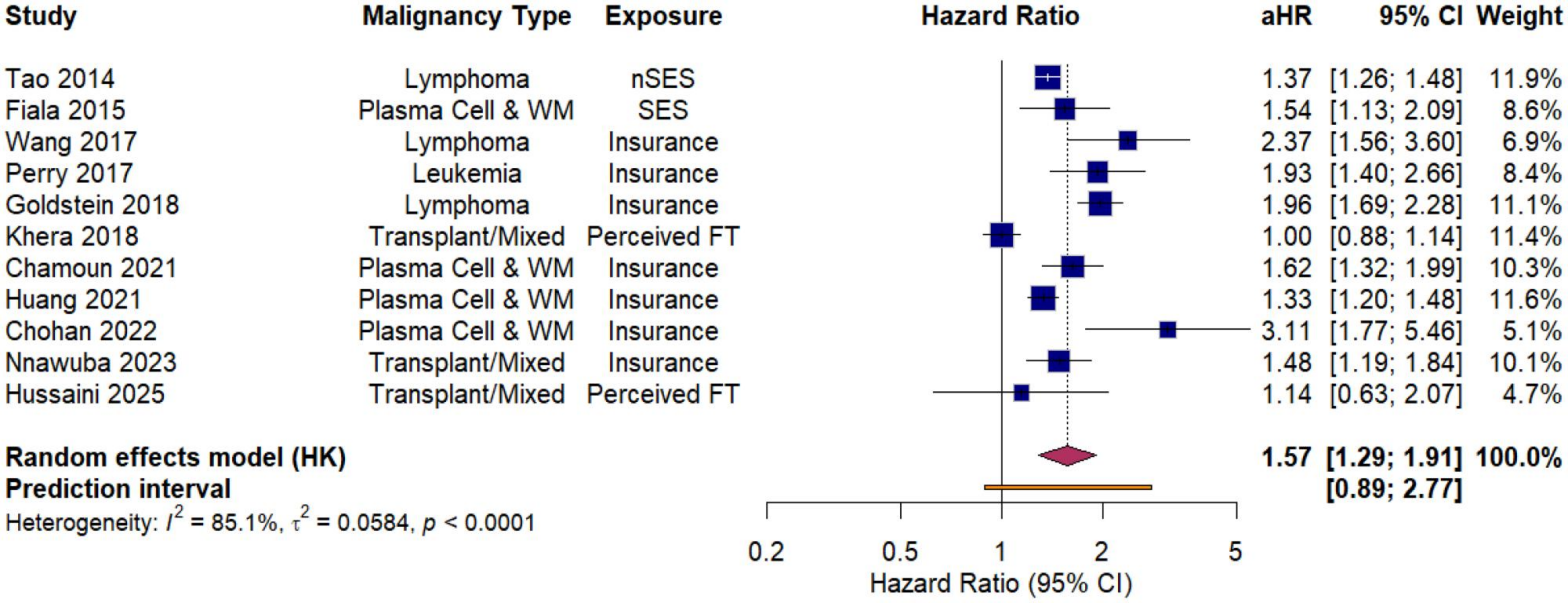
Forest plot illustrating the association between financial toxicity and mortality in individuals with hematologic malignancies.

As synthesized in the primary meta-analysis of 11 independent cohorts (Figure 2), financial toxicity was significantly associated with inferior survival among individuals with hematologic malignancies (pooled *aHR* 1.57; 95% *CI* 1.29-1.91; *P *< .001). This prognostic link remained consistent under the Hartung–Knapp adjustment. However, the 95% prediction interval (0.89-2.77) crosses the null value, suggesting that the survival disparity may be context-dependent. The high heterogeneity observed (*I*^2^ = 85.1%, *P *< .001) further indicates that the strength of this observed association varies according to the specific malignancy subtype, the measurement metric employed, and the clinical environment. Clinically, the attenuated correlation observed in settings with high-intensity psychosocial support suggests that institutional structures may mitigate the survival risks associated with economic strain.

Adjusted hazard ratios (aHRs) from 11 cohorts were pooled using a random-effects model with Hartung–Knapp (HK) adjustment. Data are stratified by financial toxicity measurement method: health insurance status, socioeconomic status (SES), and subjective patient-reported outcome measures (PROMs). For each study, the blue squares and horizontal lines represent the aHR and its corresponding 95% confidence interval (CI); square size is proportional to the study’s weight. The diamond denotes the overall pooled aHR, and the solid orange bar at the bottom indicates the 95% prediction interval. The test for subgroup differences (*P *< .001) indicates that the association between financial toxicity and mortality varies significantly across different quantification frameworks, with the most pronounced risks observed in cohorts defined by insurance status.

### Subgroup analyses by malignancy type and clinical setting

**Figure 3.**
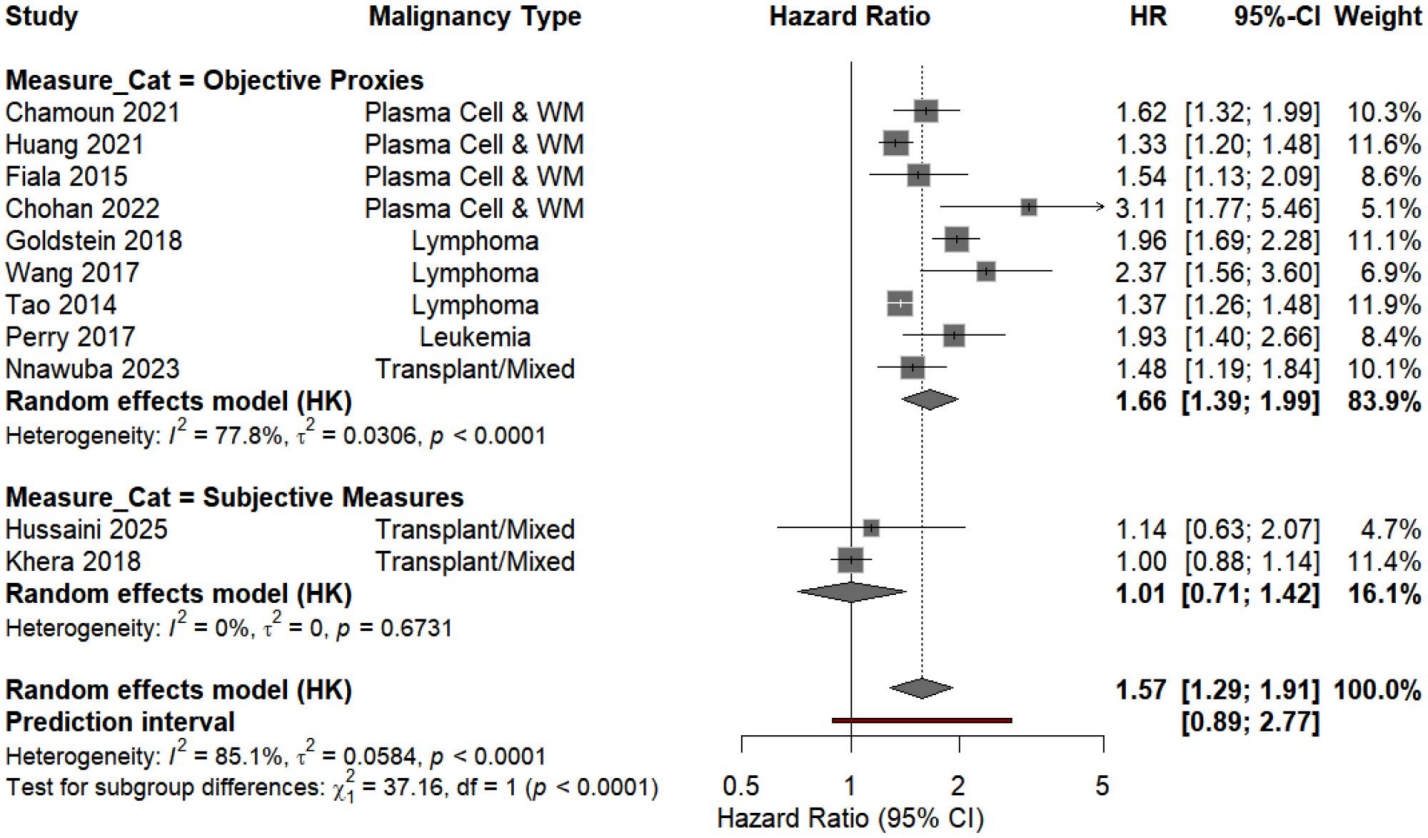
Subgroup analysis of mortality risk stratified by the definition and measurement proxies of financial toxicity.

Subgroup analysis by financial toxicity quantification methodology revealed a divergence between objective and subjective measures (Figure 3). In studies utilizing objective socioeconomic proxies, financial toxicity was associated with a higher risk of mortality (pooled *aHR*: 1.66; 95% *CI*: 1.39-1.99; *I*^2^ = 77.8%). Conversely, no significant association was observed in studies employing subjective patient-reported outcome measures (pooled *aHR*: 1.01; 95% *CI*: 0.71-1.42; *I*^2^ = 0%). Notably, this subjective measures subgroup exclusively comprised individuals within hematopoietic cell transplantation programs.

To address potential study-design bias, a sensitivity analysis was performed on prospective cohorts (*k* = 2; Hussaini et al. and Khera et al.). Similar to the subjective-measure subgroup, this analysis showed no significant association between financial toxicity and mortality (pooled HR = 1.01; 95% CI: 0.89-1.14; *P *= .93; Supplementary Figure S1), with zero statistical heterogeneity (*I*^2^ = 0.0%, *P *= .67). These results suggest that the observed correlation between financial toxicity and survival outcomes may be attenuated in prospective settings, potentially relating to the rigorous clinical monitoring and integrated supportive care inherent to such designs.

Collectively, these findings indicate that the mortality correlation is most pronounced when financial toxicity acts as structural barriers. The lack of prognostic signal in transplant cohorts may reflect the mitigating effects of embedded social support and financial navigation services in high-intensity care environments.

The association between financial toxicity and mortality was evaluated across two primary assessment frameworks: objective proxies (insurance status and neighborhood socioeconomic status) and subjective patient-reported outcome measures (COST score and financial hardship surveys). For each subgroup, diamonds represent the pooled adjusted hazard ratio under a random-effects model with Hartung–Knapp adjustment. Statistical heterogeneity within each subgroup is reported via the *I*^2^ statistic. The test for subgroup differences (*P *< .001) indicates that the prognostic signal of financial toxicity is significantly modulated by the metric employed and the associated clinical context.

### Sensitivity analysis

To assess the influence of methodological quality and publication type, studies were stratified by Newcastle-Ottawa Scale scores (Supplementary Figure S2). In the high-quality subgroup (n = 8 cohorts, NOS 8-9; all peer-reviewed), the association between financial toxicity and survival remained consistent (pooled HR: 1.56; 95% *CI*: 1.21-2.01; *P *< .001). The moderate-quality subgroup (n = 3 cohorts, NOS 6-7; all conference abstracts) yielded a comparable estimate (*HR*: 1.62; 95% *CI*: 0.69-3.79), although with a wider confidence interval crossing the null. No significant difference was observed between these strata (*P* for subgroup difference = .86). This consistency indicates that the primary pooled estimate is robust and not driven by lower-quality evidence.

### Publication bias and small-study effects

**Figure 4.**
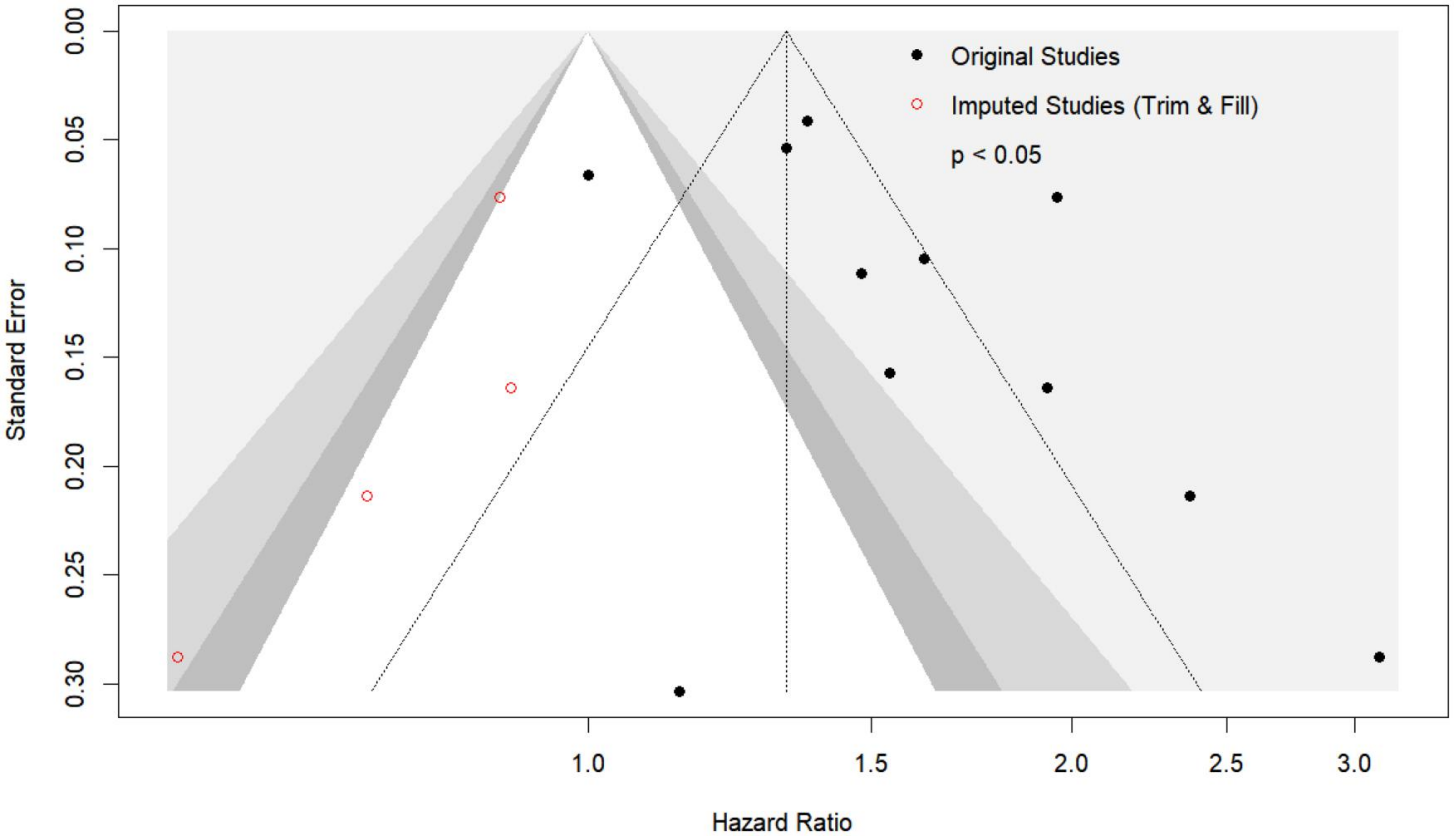
Contour-enhanced funnel plot for the assessment of small-study effects and publication bias.

Potential publication bias and small-study effects were evaluated using a contour-enhanced funnel plot (Figure 4). Egger’s test indicated significant asymmetry (*t *= 2.97, *df *= 10, *P *= .0139). To address this, the nonparametric Duval and Tweedie trim-and-fill method was performed, identifying 4 potentially missing studies. Imputing these studies yielded an adjusted pooled hazard ratio of 1.33 (95% *CI*: 1.05-1.69, *P *= .0226). The association between financial toxicity and mortality remained statistically significant after this conservative adjustment, confirming the stability of the primary findings.

The observed asymmetry should be interpreted in the context of high clinical heterogeneity (*I*^2^ = 85.1%), which may drive funnel plot asymmetry independent of publication bias. In our cohort, this asymmetry is largely attributable to smaller studies in specialized transplant settings (eg, Khera et al. and Hussaini et al.) reporting null associations, contrasting with the higher ratios observed in other cohorts. Consequently, while the unadjusted HR (1.57) may represent an upper-bound estimate, the adjusted analysis (HR 1.33) provides a conservative baseline, reaffirming that financial toxicity is significantly associated with inferior survival in hematologic malignancies.

The standard error of the log hazard ratio is plotted against the observed hazard ratio for each included cohort. Background shading delineates regions of statistical significance (*P *< .05, .05 < *P * <  .1, and *P  *>  .1). Visual inspection and Egger’s linear regression test (*P *= .0139) indicated significant asymmetry. The distribution pattern suggests that this asymmetry is primarily driven by underlying clinical heterogeneity (eg, smaller, highly monitored transplant cohorts reporting null effects) rather than systematic nonpublication of negative findings.

## Discussion

Our findings suggest a significant association between economic strain and inferior survival, with an approximately 50% increase in mortality risk observed across diverse cohorts of individuals with hematologic malignancies. However, our subgroup and sensitivity analyses reveal that this prognostic link is not uniform across all care settings. The association with mortality is most pronounced when financial toxicity manifests as structural barriers, such as a lack of insurance, particularly in conventional hematology clinics. In contrast, this association is markedly attenuated within specialized transplant programs that integrate proactive financial navigation into standard care. These data suggest that financial toxicity is a critical factor relevant to overall survival, extending beyond its established impact on quality-of-life.

### Structural factors vs psychosocial burden

The pronounced heterogeneity observed in our analysis (*I*^2^ = 85.1%) appears related to the operationalization of financial toxicity. Objective measures, such as uninsured status or Medicaid enrollment, were consistently associated with heightened mortality (pooled aHR: 1.66). This likely reflects structural barriers, where inadequate coverage may correlate with delayed diagnoses, limited treatment selection, or inconsistent access to high-cost continuous therapies, such as novel targeted agents or immunotherapies. In contrast, while subjective measures (eg, the COST tool) capture the psychosocial and behavioral burden of out-of-pocket costs, our data indicate that perceived financial hardship may not inherently compromise survival if patients maintain access to the healthcare system.

### Mechanisms linking economic strain to outcomes

The impact of financial toxicity is often compounded by the cumulative burden of prolonged therapy. In multiple myeloma and lymphoma, as individuals progress through successive treatment lines, medical costs escalate while treatment-free intervals shrink.^27^ This suggests that financial toxicity is an evolving stressor that tracks with disease advancement. Furthermore, psychological distress may act as a mediator; economic stress has been linked to reduced treatment adherence and eroded well-being.^28^ Together, these findings suggest a framework where the observed mortality risks are associated with both overt treatment avoidance and the gradual compromise of adherence as economic burdens mount.

### The institutional buffering effect in transplantation

The lack of a significant mortality association within the hematopoietic cell transplantation subgroup (pooled aHR: 1.01) provides a critical insight into the role of institutional support. Unlike conventional outpatient hematology, transplant programs often integrate rigorous psychosocial evaluations, financial navigation, and dedicated social work. These systemic interventions may ensure that even when patients report high financial toxicity, their access to critical post-transplant care and immunosuppressive is maintained. The survival parity observed in these cohorts suggests that the correlation between financial toxicity and mortality may be mitigated through systemic interventions. This hypothesis is further supported by our sensitivity analysis restricted to prospective studies (HR 1.01, *P *= .93), which were predominantly conducted in tertiary hematopoietic cell transplantation centers. In these environments, embedded support structures may attenuate the link between subjective financial hardship and survival outcomes. While financial toxicity remains a dynamic risk due to complications such as graft-vs-host disease, the contrast between hematopoietic cell transplantation and general hematology settings highlights that the prognostic risk associated with economic strain is modifiable. Expanding proactive financial navigation models into broader clinical practice could potentially address the survival disparities observed in nontransplant settings.

### Methodological considerations and limitations

Several limitations of this meta-analysis should be acknowledged. Most notably, the reliance on retrospective data introduces potential for unmeasured confounding. While most included studies adjusted for available demographic and disease-specific variables, it is difficult to isolate the independent effect of financial toxicity in retrospective datasets. Financial strain is rarely the sole factor contributing to inferior mortality; instead, it often coexists with unmeasured clinical confounders such as baseline performance status, detailed cytogenetic risk, severity of comorbidities, or specific treatment adherence. These interwoven factors may independently drive both the economic burden and the worsening survival outcomes, precluding any definitive causal inferences. Furthermore, retrospective registry data often lack granular details on cytogenetic risk or treatment adherence, potentially confounding the observed link between financial status and survival.

Methodologically, the identified funnel plot asymmetry (*P *= .014) suggests that the pooled estimate should be interpreted with caution. Although the trim-and-fill method yielded a more conservative association, the consistency of the findings suggests a robust underlying correlation. Additionally, proxy measures like insurance status may not capture the full spectrum of personal asset depletion. In transplant settings, patient-reported measures are also susceptible to survivorship and immortal time biases, as the most financially vulnerable individuals may fail to reach the transplant stage or survey completion. Finally, as the evidence base is predominantly US-centric, the pronounced mortality correlation observed in the single Indian cohort^25^ from a low-resource setting highlights the urgent need for further large-scale prospective data outside the US healthcare systems. While we addressed heterogeneity through subgroup analyses, the biological diversity across different hematologic malignancies remains a potential confounding factor that warrants further exploration in disease-specific prospective cohorts.

## Conclusion

Financial toxicity is associated with worse survival in individuals with hematologic malignancies, particularly when identified through structural barriers such as inadequate insurance coverage. This observed correlation was markedly attenuated in clinical settings characterized by integrated supportive services and financial navigation, suggesting that the impact of economic strain on survival may be a modifiable factor. These findings underscore a clinical mandate to move beyond the mere recognition of financial toxicity toward the systematic integration of financial screening and navigation into standard oncology care pathways. Addressing these survival disparities is essential to ensure that clinical advancements translate into equitable outcomes across all socioeconomic strata.

## Supplementary Material

oyag178_Supplementary_Data

## Data Availability

The data that support the findings of this study are available from the corresponding author upon reasonable request.
