# Supplementary figures and images for: The association between financial toxicity and mortality in hematologic malignancies: a systematic review and meta-analysis
Source: Oncologist. 2026 May 7;31(7):oyag178. doi: 10.1093/oncolo/oyag178 (PMC13242265; doi:10.1093/oncolo/oyag178)

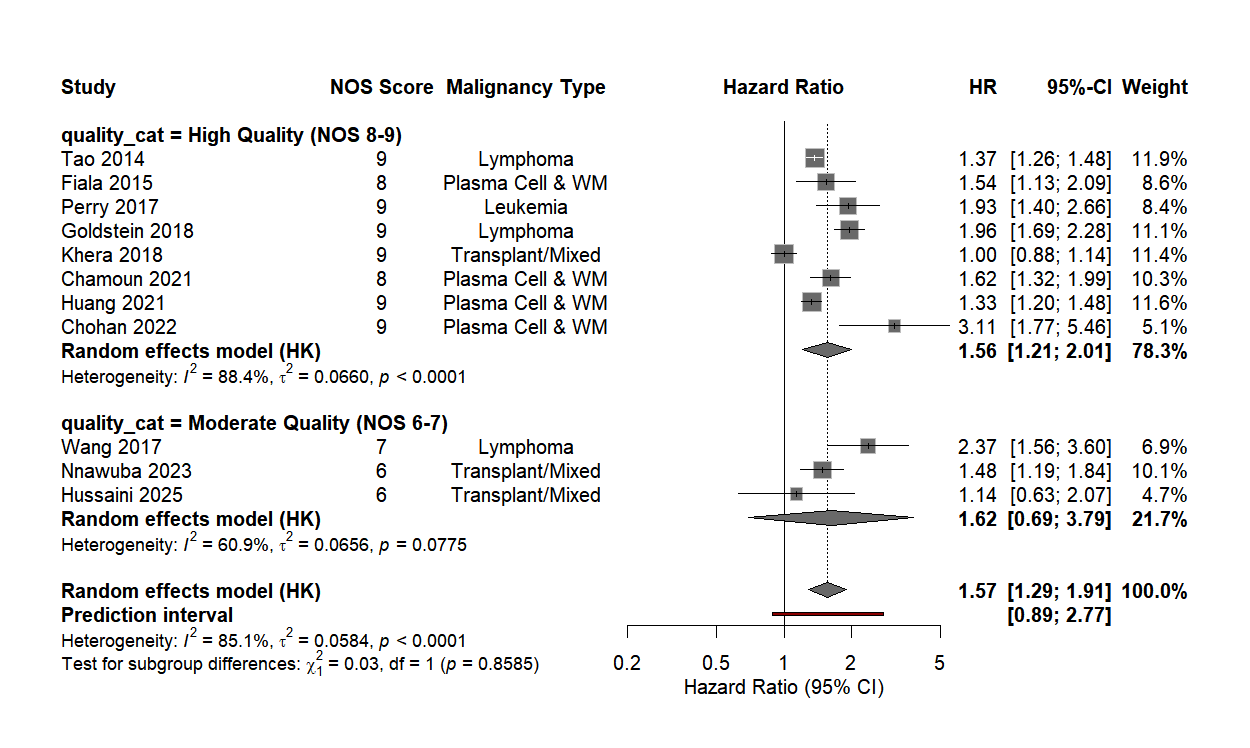

Supplement: oyag178_Supplementary_Data [file oyag178_supplementary_data.zip › Figure.S2.tiff]

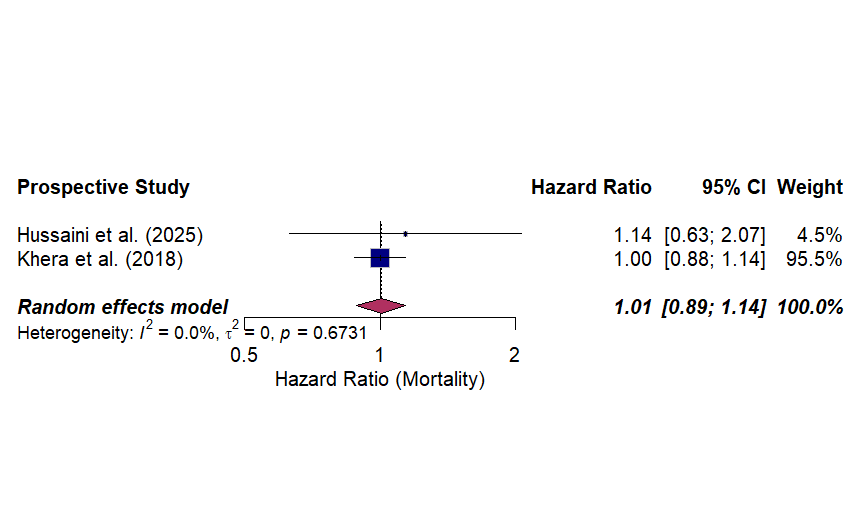

Supplement: oyag178_Supplementary_Data [file oyag178_supplementary_data.zip › Figure S1.tiff]
